# Rethinking trauma in an age of total visibility

**DOI:** 10.3389/fpsyt.2026.1735379

**Published:** 2026-09-23

**Authors:** Noa Herz

**Affiliations:** Department of Neurology and Neuroscience, Vickie and Jack Farber Institute for Neuroscience, Sidney Kimmel Medical College, Thomas Jefferson University, Philadelphia, PA, United States

**Keywords:** diagnostic criteria, digital health, policy & disabilities, PTSD - posttraumatic stress disorder, trauma

## Abstract

The digital age has transformed the way we experience and respond to traumatic events. Continuous and vicarious exposure to distressing visual content through social media and online news has created new pathways for psychological harm that existing diagnostic frameworks fail to capture. This perspective article examines how digital technologies have blurred the boundaries between direct and indirect trauma, enabling mediated exposure to evoke stress responses comparable to those arising from firsthand experience. Using the October 7, 2023, attacks in Israel as a case study, it explores how the online circulation of graphic material has expanded the reach of trauma, reshaped its mechanisms, and challenged established clinical definitions. Building on the historical evolution of posttraumatic stress disorder, the article calls for new conceptual, technological, and clinical frameworks to address digital trauma - a form of exposure defined by its pervasiveness, accessibility, and social diffusion.

## Introduction

The human experience has always carried the dual capacity for profound empathy and heartbreaking cruelty. Yet, in the digital era, it is often the echoes of cruelty that resonate most widely; Traumatic events now circulate online at unprecedented speed and scale, confronting vast audiences with sights once reserved for a limited number of direct witnesses. The attacks of October 7, 2023, in Israel revealed this transformation with devastating clarity. Civilians were abducted, terrorized, and murdered in acts of extreme violence, while these acts were broadcast through digital platforms. Videos, livestreams from the killing fields, and desperate messages of those trapped in their homes spread instantaneously across social media, bypassing psychological defenses and entering collective consciousness with visceral immediacy.

The digital era has not only changed how we document violence; it has changed how we experience it. Trauma became a continuous and socially distributed experience. Individuals far removed from physical danger may nonetheless experience the mental and emotional toll of trauma, marked by intrusive imagery, hyperarousal, avoidant behaviors, and cognitive and mood deterioration. Our diagnostic frameworks, still anchored to discrete, proximal events, are ill-equipped to capture this form of exposure that blurs the boundary between witness and victim.

## From denial to recognition

The struggle to define trauma has always mirrored the cultural anxieties of its time. In the early 20th century, psychoanalytic thought shaped psychiatry’s understanding of trauma, seeking explanations for suffering within the psyche rather than the outer world. Freud’s retreat from the Seduction Theory, which linked neuroses to actual childhood abuse, signaled a shift from external events to internal conflicts. Trauma, in this framework, became less about what happened to a person and more about how they symbolically processed it. The external world was demoted; the patient’s inner world became the focus.

These ideas carried through mid-century psychiatry. The first two editions of the *Diagnostic and Statistical Manual of Mental Disorders* (DSM) reflected this stance: trauma-related conditions were framed as transient “situational disturbances,” expected to resolve quickly. Even the suffering of soldiers returning from Korea and Vietnam - plagued by flashbacks, nightmares, and rage - was often pathologized as moral weakness or maladjustment rather than the consequence of extreme experiences. The 1980 inclusion of posttraumatic stress disorder (PTSD) in DSM-III finally recognized that external events could leave enduring psychological scars. However, every diagnostic advance has come with its own blind spots. The 9/11 attacks, for instance, expanded the field’s understanding of indirect trauma, when people across the world developed stress reactions after watching live footage of the collapsing towers. The COVID-19 pandemic later introduced a different form of prolonged, diffuse, anticipatory trauma. October 7 events in Israel now add another inflection point: the traumatic events were not only mediated through the media, but were an active, deliberate weaponization meant to inflict mental harm. The live-stream videos were not byproducts; they were an inherent part of the terrorists’ attack.

The evolution of trauma theory has increasingly recognized that psychological responses cannot always be understood through a single, discrete traumatic event. Herman’s work on complex trauma emphasized the effects of prolonged and repeated exposure, particularly when trauma is interpersonal and inescapable (1). This perspective contributed to the later recognition of complex posttraumatic stress disorder (C-PTSD), which extends beyond the core symptoms of PTSD to include persistent alterations in emotion regulation, self-perception, and relationships (2). C-PTSD offers an important conceptual precedent for thinking about trauma that unfolds through repeated exposure rather than a single event. Yet digital exposure presents a different challenge: the individual may be geographically removed from the original event while repeatedly encountering its images, testimonies, and aftermath. Whether such mediated exposure can produce the broader disturbances associated with complex trauma remains an empirical question.

## Digital exposure as psychological reality

The new disturbing realities of unfiltered social media underscore a widening gap between our formal definitions of trauma and the lived experiences of the digital age. According to DSM-V, a diagnosis of PTSD still hinges on a proximal event - direct or witnessed exposure to death, serious injury, or sexual violence (3). Indirect exposure may qualify, but only under narrowly defined conditions (4), usually limited to professionals repeatedly exposed to details of trauma in their work. This rigid boundary, which determines diagnostic eligibility, has powerful implications - not just for diagnostic clarity, but for moral legitimacy and personal meaning. When GoPro cameras document the torture of your loved ones, as happened in the October 7^th^ events, the geographical distance has little bearing on the inflicted emotional toll. History has already taught us the harm in dismissing the long-term effects that external stressors can have on mental health. We’ve come a long way from the days when symptomatic war veterans were treated as weak or treasonous (5), toward an understanding that everyone has a breaking point. Yet a lingering fear remains: that broadening the criteria for PTSD risks diluting its meaning or opening the door to claims tied to compensation (6). This concern continues to shape our clinical thinking around a mental health condition that should remain uncontaminated by economic considerations.

The caution in expanding what qualifies as a traumatic event, though grounded in the desire to protect the integrity of the diagnosis, now risks stalling clinical progress. Research shows that exposure to traumatic events through media can produce psychological effects as severe as, and at times worse than, those resulting from direct exposure (7, 8). Symptoms intensify the longer one watches the disturbing content (9, 10), reflecting a form of cumulative stress unique to the digital environment. Traumatic content tied to mass events spreads pervasively across platforms, making avoidance nearly impossible.

Perceived sense of control, a central predictor of the development of long-term stress reactions (11), reaches its lowest point when individuals witness harm inflicted on loved ones unfolding on their screens. Children and adolescents, in particular, are highly vulnerable to the psychological impact of such unfiltered content and may bear the deepest and most lasting scars from this emerging form of trauma (12).

## Toward a new framework of digital trauma

If the 20th century taught us that trauma is not a sign of character weakness, the 21st century must teach us that trauma is no longer bound by geography. The challenge before us is not merely to expand diagnostic definitions but to rethink the very notion of trauma exposure. This is not a call to dilute the concept of trauma to include all distress, but rather to ensure that our frameworks reflect the realities of modern life. To cling to outdated definitions is to repeat the mistakes of the past, to once again deny recognition from those who suffer.

The field now faces urgent empirical and theoretical questions: How do we assess and diagnose stress-related disorders when the boundaries between direct and indirect exposure, primary and secondary trauma, have blurred? How do we contend with digital archives of traumatic events that are endlessly accessible, searchable, and recirculated? And what are the psychological consequences of memories that are documented, stored, and permanently retrievable on our personal devices?

The answers to these questions extend beyond the clinic, reaching into the realms of technology and public policy. Media outlets, driven by the inherent imperative to capture attention, must be held accountable for the societal cost of broadcasting distressing graphic content. Social media platforms should adopt protective features - dynamic buffering tools such as delayed autoplay, intensity-scaled content warnings, and mental health alerts for users exposed to large volumes of traumatic material. Public health agencies must create screening protocols for high-exposure groups and invest in digital trauma clinics that combine telehealth with community outreach. Clinically, we need updated tools that assess digital exposure and offer therapeutic strategies that prioritize media hygiene.

## Conclusions

A century ago, the greatest challenge in the stress-related psychiatry field was persuading the world that trauma was real - that psychic wounds could be as devastating as physical ones. Today, the challenge is recognizing that such wounds can be inflicted not only by what we experience firsthand, but also by what we witness, replay, and share through our digital devices. The digital age has collapsed the distance between observer and victim.

The October 7 attacks in Israel may represent one of the most far-reaching, unintentional experiments in mediated trauma exposure in human history. Its long-term consequences - for survivors, for Israeli society, and for how we understand and treat trauma - are still unfolding. But the urgency is already clear: we need new frameworks - clinical, ethical, and technological -to grasp and respond to trauma in an era of total visibility. We need to better understand the line between documentation and re-traumatization, between bearing witness and being digitally shackled to a moment of horror.

Our diagnostic frameworks, designed for 20th-century warfare and natural disasters, are poorly suited to an era of total visibility. To meet this moment, we must redefine trauma to include mediated, continuous exposures and develop clinical and policy tools that reflect the realities of modern psychological harm.

## Data Availability

The original contributions presented in the study are included in the article/supplementary material. Further inquiries can be directed to the corresponding author.
